# Targeting an engineered cytokine with interleukin-2 and interleukin-15 activity to the neovasculature of solid tumors

**DOI:** 10.18632/oncotarget.27772

**Published:** 2020-11-03

**Authors:** Michael R. Mortensen, Jacqueline Mock, Marco Bertolini, Marco Stringhini, Marco Catalano, Dario Neri

**Affiliations:** ^1^Department of Chemistry and Applied Biosciences (D-CHAB), Institute for Pharmaceutical Sciences (IPW), 8093 Zurich, Switzerland

**Keywords:** immunotherapy, immunocytokines, EDB of fibronectin, antibody-cytokine fusion proteins, engineered cytokine

## Abstract

There is a growing interest in the antibody-based delivery of cytokines to the tumor environment as a means to boost the anti-cancer activity of tumor-resident T cells and NK cells. Here, we describe the expression and characterization of fusion proteins, featuring the L19 antibody (specific to the alternatively-spliced EDB domain of fibronectin) and an engineered cytokine with interleukin-2 and interleukin-15 properties. The cytokine moiety was fused either at the N-terminal or at the C-terminal extremity and both fusion proteins showed a selective tumor accumulation in a quantitative biodistribution experiment. The N-terminal fusion inhibited tumor growth in immunocompetent mice bearing F9 carcinomas or WEHI-164 sarcomas when used as single agent. The anticancer activity was compared to the one of the same cytokine payload used as recombinant protein or fused to an anti-hen egg lysozyme antibody, serving as negative control of irrelevant specificity in the mouse. These results indicate that the antibody-based delivery of engineered cytokines to the tumor neovasculature may mediate a potent anticancer activity.

## INTRODUCTION

Cancer immunotherapy relies on the activation of certain leukocytes (most typically, T cells and/or natural killer cells), with the aim to induce a selective biocidal activity against tumor cells. Immunotherapy approaches, which may include the use of recombinant cytokines [1–4], immune checkpoint inhibitors [5–7] and immune cells [8, 9], are increasingly being used in the clinical practice. In 1992, interleukin (IL)-2 was approved as the first cancer immunotherapy, providing a long-term survival benefit to a small proportion of patients with metastatic melanoma or renal cell carcinoma [10–12]. However, high-dose IL2 causes substantial toxicity, thus limiting this treatment modality to younger and physically fit patients. The modest accumulation of IL2 at the site of disease [13] and its short half-life [14, 15] represent additional limitations for a broader applicability of this immunostimulatory agent in cancer treatment.

Recently, there has been a growing interest in the engineering of novel cytokine products with improved anticancer properties and better tolerability. Molecular strategies have included the generation of antibody fusion proteins, the introduction of mutations in the cytokine moiety and/or the conjugation to polymers [3, 16–27]. IL2 muteins may alter the interaction of the cytokine with one or more of the IL2 receptor subunits [20], thus altering the selectivity towards the intermediate affinity receptor [consisting of CD122 (IL2Rβ) and CD132 (γ_C_)] or towards the high affinity receptor [consisting of CD25 (IL2Rα), CD122, and CD132] [28]. Since regulatory T cells (Tregs) predominantly express the high affinity receptor [28], the selective activation of the intermediate affinity receptor (expressed by resting and memory lymphocytes including CD8^+^ T cells) [28] has attracted considerable research efforts [29–35]. In one approach, the strength of the interaction of IL2 with the cognate intermediate affinity receptor was increased through mutations, which also stabilized folding [34]. Treating tumor-bearing mice with such a mutein expanded both Treg and CD8^+^ T cells to a lesser extent than memory CD8^+^ T, compared to the administration of wild-type IL2. The increased CD8^+^: Treg ratio correlated with an improved therapeutic effect [34]. In a second approach, an increased selectivity towards the intermediate affinity IL2 receptor was achieved by blocking the interaction with CD25 either through IL2 mutations or by antibody blockade of the IL2 epitope involved in CD25 binding [31–33, 35]. Interestingly, a decreased IL2 toxicity was observed in CD25 knock-out mice [36].

In 2019, Silva *et al.* described a series of engineered cytokines, which displayed biological features intermediate between those of IL2 and of IL15. IL15 interacts with CD122 (IL2Rβ) and CD132 (γ_C_) like IL2, but recognizes a different alpha subunit (CD215, rather than CD25). One of the new computationally designed cytokine variants, termed by the authors Neoleukin™-2/15, exhibited an increased affinity towards the receptor components shared by IL2 and IL15, with a complete absence of CD25 binding. Moreover, the new engineered cytokine was much more stable compared to wild-type IL2 [37]. Treatment of mice with recombinant preparations of the cytokine mimetic led to an increased CD8^+^: Treg ratio and improved therapeutic effects compared to murine IL2 in mouse cancer models, suggesting that the new cytokine may be more suitable for pharmaceutical applications. However, the protein has a small size (approximately 12 kDa), clears rapidly from circulation and lacks a tumor targeting moiety.

While the half-life extension of IL2 has been addressed by PEGylation [21] and albumin-fusion [38], selective tumor accumulation has generally been achieved by fusion to antibodies forming “immunocytokines” [3, 16]. The choice of a suitable antibody format may heavily influence biodistribution properties and performance [16, 39, 40]. Antibody fragments are cleared more rapidly than full-length IgG’s and may lead to superior tumor: blood ratios *in vivo* [16]. Fusion proteins based on the L19 antibody (specific to the alternatively-spliced extra-domain B (EDB) of fibronectin, a marker of angiogenesis) and on IL2 or TNF are being investigated in Phase III clinical trials for the treatment of patients with melanoma (https://clinicaltrials.gov/ NCT02938299, EudraCT 2015–002549-72) or with soft-tissue sarcoma (https://clinicaltrials.gov/ NCT03420014, EudraCT 2016-003239-38) [41]. The EDB of fibronectin represents an attractive tumor-associated antigen, which is expressed in most malignancies but is undetectable in most normal tissues except for some structures in female reproductive organs [42].

In this work, we have fused the L19 antibody in diabody format to Neoleukin™-2/15 (termed “L19-Neo™” and “Neo™-L19”). The new fusion proteins were able to selectively localize at the tumor site (as evidenced by quantitative biodistribution experiments with radiolabeled protein preparations). Neo™-L19 displayed a potent anticancer activity in two immunocompetent mouse models of cancer.

## RESULTS

### Cloning, expression and *in vitro* characterization of fusion proteins

**Figure 1:**
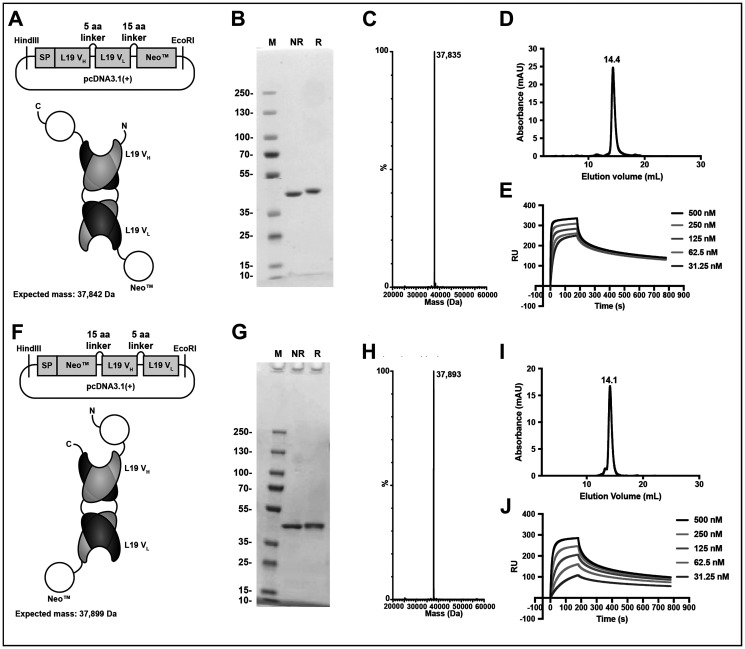
Design and *in vitro* characterization of the immunocytokine fusion proteins. Top, L19-Neo™. Bottom, Neo™-L19. (**A**, **F**) Cloning scheme, expected protein structure and expected mass. (**B**, **G**) SDS-PAGE analysis stained for protein by Coomassie Blue. M: PageRuler™ Plus Prestained Protein Ladder, NR: non-reducing, R: reducing. (**C**, **H**) MS profile. (**D**, **I**) Size exclusion chromatography analysis. (**E**, **J**) Surface plasmon resonance experiment using a serial dilution of the fusion protein and surface immobilized EDB of fibronectin.

The engineered cytokine Neoleukin™-2/15 moiety was genetically fused at the N- or C-terminus of the L19 antibody in diabody format (Figure 1A and Supplementary Sequence Information). The linker connecting the two moieties comprised 15 amino acids (Figure 1A, 1F), while a short linker (5 amino acids) between V_H_ and V_L_ ensured a stable diabody formation [43]. The fusion proteins (termed L19-Neo™ and Neo™-L19) were expressed in CHO cells and purified by protein A affinity chromatography, utilizing the engineered protein A binding site on L19 [44]. The molecular weight of the fusion proteins was confirmed by LC/MS. SDS-PAGE and size exclusion chromatography analyses indicated a high purity and homogeneity (Figure 1). Avid binding of the fusion proteins to EDB was confirmed by surface plasmon resonance (Figure 1E, 1J).

**Figure 2:**
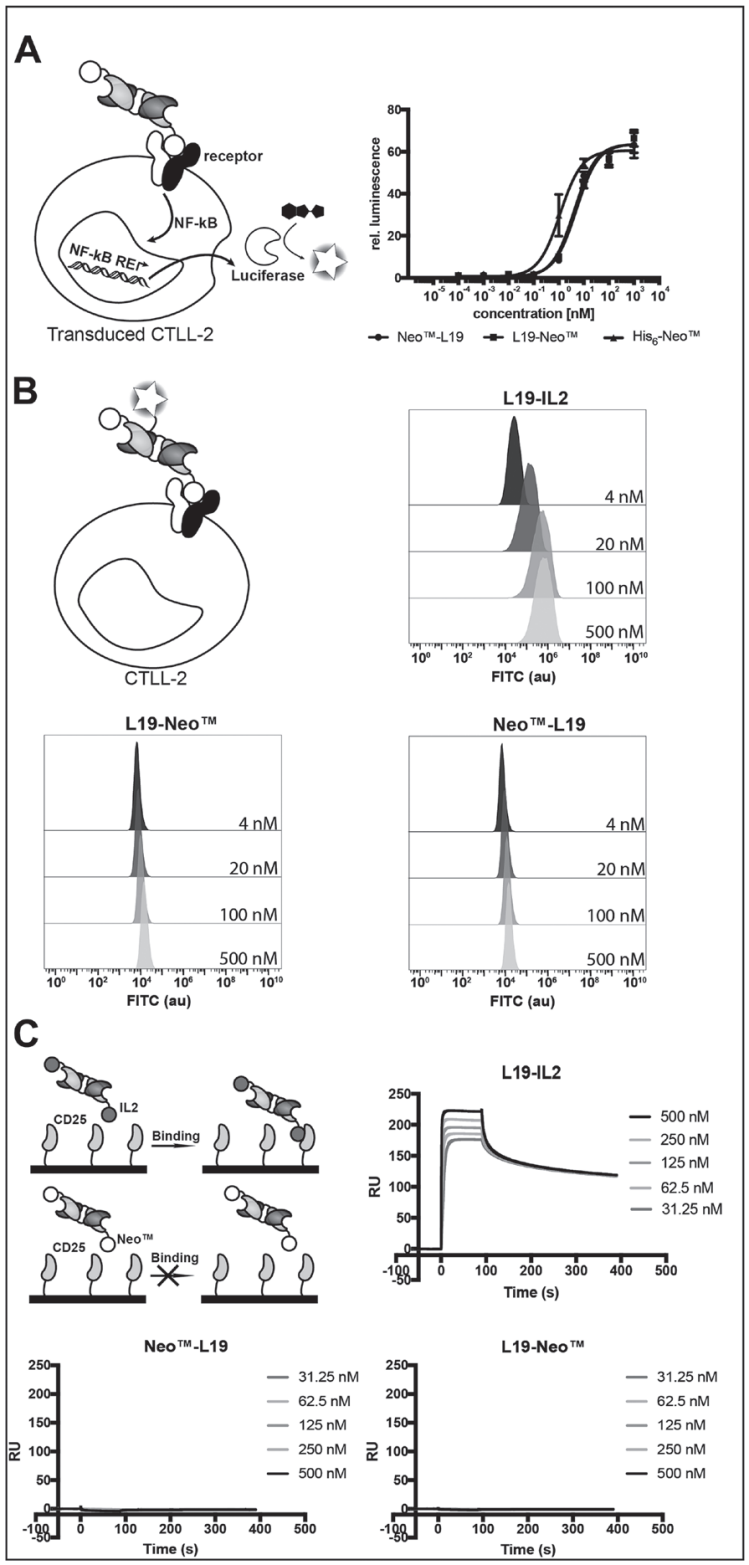
Characterization of the cytokine moiety in the fusion proteins. (**A**) NF-κB coupled luciferase reporter assay comparing the fusion proteins with His_6_-Neo™. EC50 (mean (*n* = 3) ± SD): L19-Neo™ 5.01 ± 0.57 nM, Neo™-L19 4.14 ± 0.55 nM, His_6_-Neo™ 1.14 ± 0.21 nM. (**B**) Binding investigating of FITC-modified fusion proteins on murine CTLL-2 cells by flow cytometry. (**C**) SPR experiment using a serial dilution of fusion protein in the solution phase and surface immobilized hCD25. Zoomed images of the sensograms for the fusion proteins are shown in Supplementary Figure 2.

We tested cytokine activity using a recently reported NF-κB reporter assay in transduced murine CTLL-2 cells [45] (Figure 2A) and a proliferation assay with murine CTLL-2 cells (Supplementary Figure 1). Both assays indicated that the fusion proteins had similar cytokine activities, which was comparable to the one of the recombinant cytokine. The binding of the cytokine moiety to its cognate receptor was investigated by flow cytometry on murine CTLL-2 cells using fluorescein-modified immunocytokines. Surprisingly, L19-Neo™ and Neo™-L19 did not show binding to the very same cells that were successfully used for the proliferation assay, while the L19-IL2 fusion protein [13], used as positive control, showed a concentration-dependent increase in average fluorescence signal, as expected (Figure 2B). The discrepancies might be a result of differences in binding kinetics, since the experiment requires a long-lasting interaction for binding to be observed. We confirmed that L19-IL2 was able to bind to hCD25 by surface plasmon resonance, while L19-Neo™ and Neo™-L19 did not interact with the receptor subunit (Figure 2C and Supplementary Figure 2).

### *In vivo* biodistribution and therapy studies

**Figure 3:**
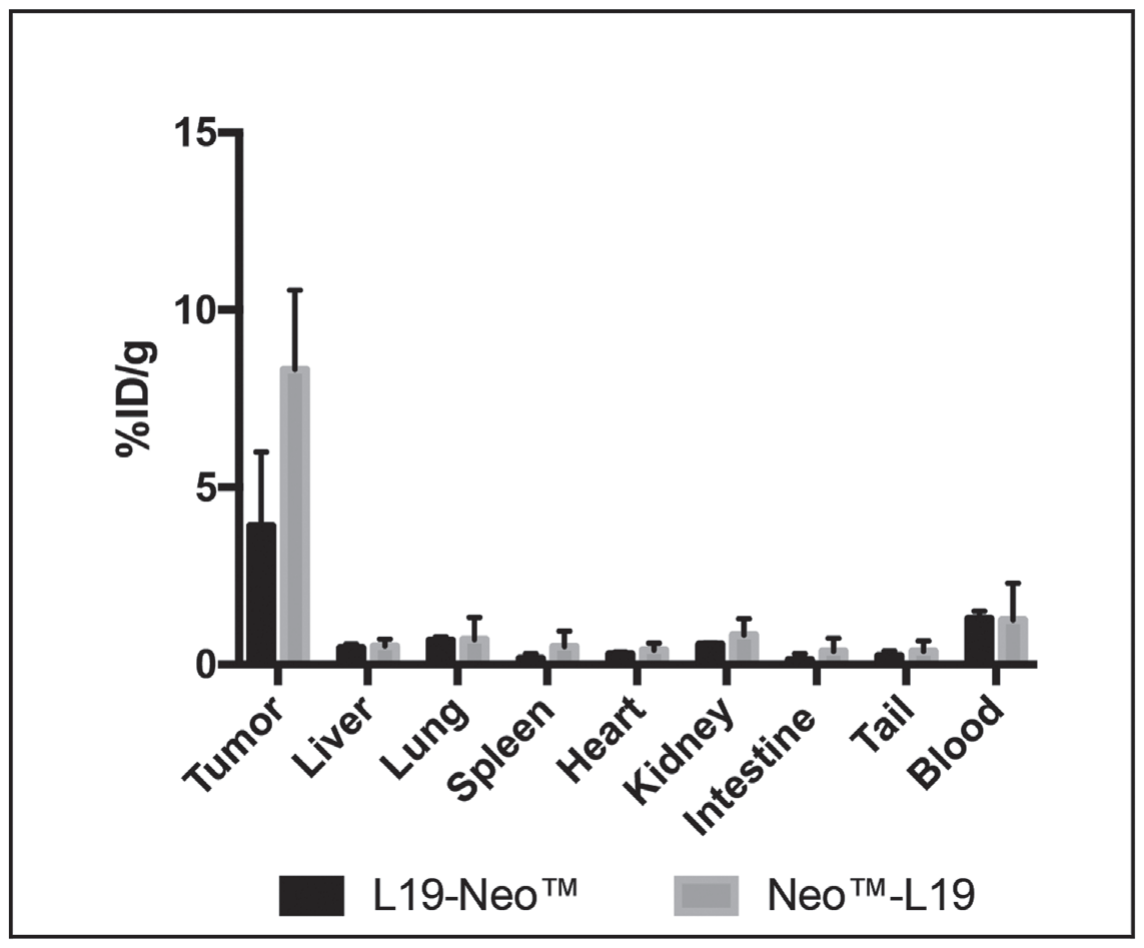
Investigation of the tumor homing potential of the fusion proteins *in vivo*. A quantitative biodistribution experiment using intravenously injected radioiodinated fusion proteins. The analysis was performed after 24 h and is expressed in percentage of the injected dose per gram of tissue (%ID/g ± SD, *n* = 3).

The tumor homing potential of the L19-Neo™ and Neo™-L19 immunocytokines was investigated by a quantitative biodistribution experiment using radiolabeled protein preparations. The products were injected intravenously into immunocompetent mice bearing F9 teratocarcinoma tumors, which were sacrificed 24 hours later. A selective tumor accumulation was observed for both fusion proteins, but Neo™-L19 showed a slightly improved percent of injected dose per gram of tumor (%ID/g; 8.3 ± 2.2% versus 3.9 ± 2.1%, respectively) and, for this reason, was chosen for subsequent therapy experiments. Both products have low levels in blood (~1%ID/g) and in other tissues (Figure 3). The biodistribution profiles were better than the ones observed using the KSF antibody moiety, specific to hen egg lysozyme and serving as negative control of irrelevant specificity in the mouse (Supplementary Figures 3 and 4).

**Figure 4:**
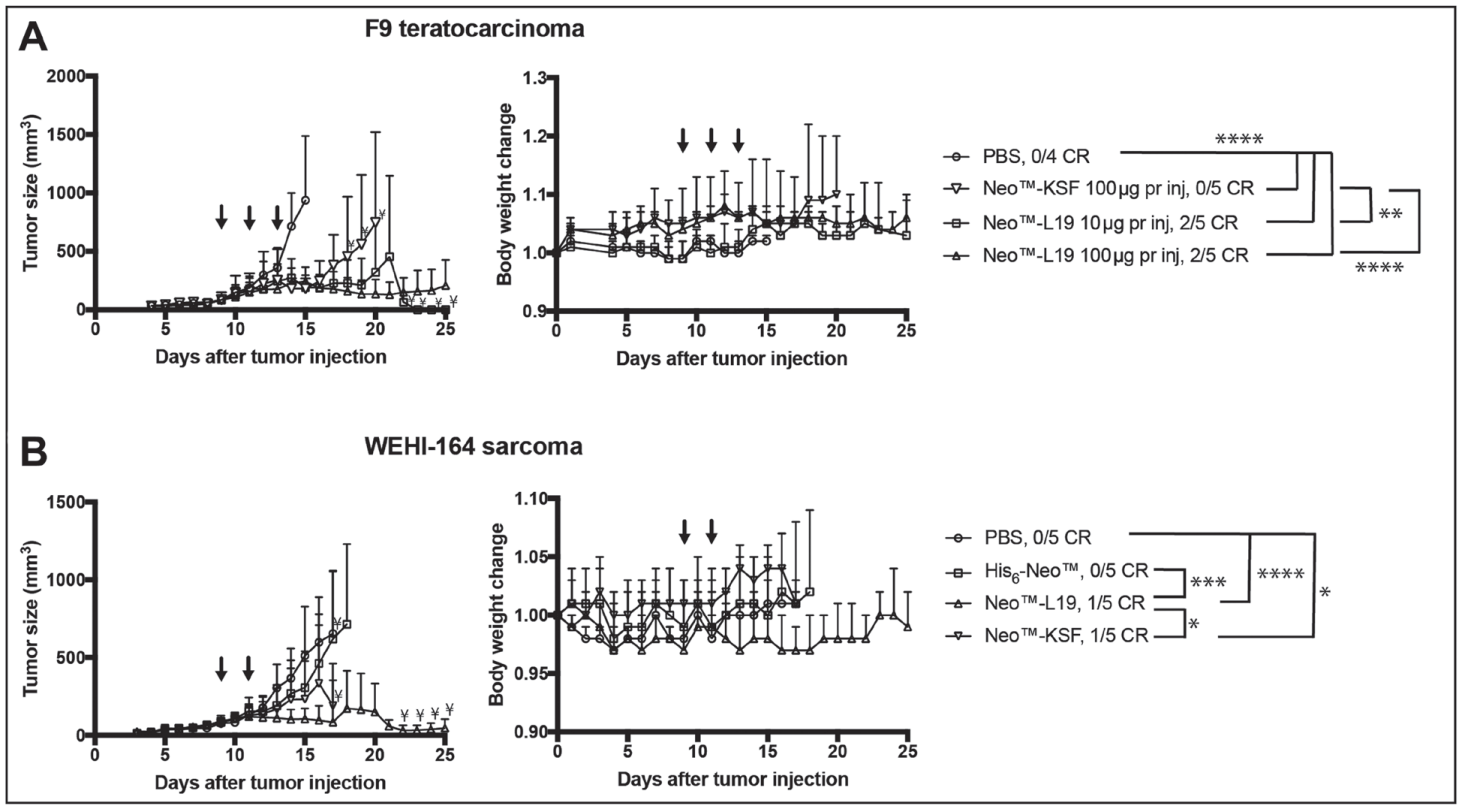
Activity of Neo™-L19 in F9 teratocarcinoma or WEHI-164 fibrosarcoma tumor bearing mice. (**A**) F9 teratocarcinoma. Left: Tumor bearing mice received 3 injections (↓) of Neo™-L19, Neo™-KSF or saline (PBS) when the tumors reached an average size of 90 mm^3^. The data is represented as the mean ± SD. Statistical analysis was performed by two-way ANOVA with post Bonferroni test (Not significant *P* > 0.05, ^*^ *P* < 0.05, ^**^ *P* < 0.01, ^***^ *P* < 0.001, ^****^ *P* < 0.0001). All the immunocytokine treatments were statistically different from the saline treatment on day 14 (^****^ *P* < 0.0001). On day 20, Neo™-L19 10 μg per inj (^**^ *P* < 0.01) and Neo™-L19 100 μg per inj (^****^ *P* < 0.0001) were different from the Neo™-KSF (100 μg per inj) group. Right: Body weight changes during the treatment represented as the mean ± SD. (**B**) WEHI-164 fibrosarcoma. Left: Tumor bearing mice received 2 equimolar injections (↓) of Neo™-L19, Neo™-KSF, His_6_-Neo™ or saline (PBS) when the tumors reached an average size of 90 mm^3^. The data is represented as the mean ± SD. On day 16, the Neo™-L19 (^****^ *P* < 0.0001) and Neo™-KSF (^*^ *P* < 0.05) groups showed a significant difference to the saline group. On day 16, Neo™-L19 was different from the His_6_-Neo™ (^***^ *P* < 0.001) and Neo™-KSF (^*^ *P* < 0.05) groups. On day 17, Neo™-L19 and Neo™-KSF was not significantly different (*P* > 0.05). Right: Body weight changes during the treatment represented as the mean ± SD. Only significant differences between treatment groups have been indicated. ^¥^only two mice remaining in the group. CR: complete response.

Therapy experiments were performed in immunocompetent mice bearing subcutaneous F9 teratocarcinomas. In an initial therapy experiment, Neo™-L19 and Neo™-KSF were administered intravenously (60 μg per injection) on days 7, 9, and 11. Both products inhibited tumor growth compared to saline treatment, but Neo™-L19 was more potent and gave a 50% cure rate (Supplementary Figure 5). In a second therapy experiment with mice bearing F9 teratocarcinomas, Neo™-L19 was injected at doses of 10 and 100 μg per injection on days 9, 11, and 13. The treatment resulted in a potent tumor-growth inhibiting activity compared to saline (Figure 4 and Supplementary Figure 6), with 2/5 durable complete responses in the Neo™-L19 group, while the nontargeted Neo™-KSF fusion did not cure any mouse (0/5). Mice with complete responses were observed for 60 days after last tumor indication, and in all cases the tumor eradication persisted. The treatments were in general well tolerated indicated by the small changes in body weights (Figure 4).

We also performed therapy experiments in immunocompetent BALB/c mice bearing subcutaneous WEHI-164 fibrosarcomas. A preliminary parallel investigation indicated that a third injection of 100 μg immunocytokine was not well tolerated, since it resulted in body weight loss (Supplementary Figure 7). Neo™-L19, Neo™-KSF and His_6_-Neo™ were administered in equimolar doses on days 9 and 11. The Neo™-L19 (^****^ *P* < 0.0001) and Neo™-KSF (^*^ *P* < 0.05) fusion proteins showed a potent tumor-growth inhibition (Figure 4 and Supplementary Figure 8), while the His-tagged recombinant cytokine, in our hands, had a modest tumor growth inhibitory effect. Collectively, the therapy experiments indicate that the tumor-homing immunocytokine Neo™-L19 potently inhibited tumor growth in two independent immunocompetent mouse models of cancer.

## DISCUSSION

High-dose IL2 is considered the first effective cancer immunotherapy, since the treatment can lead to complete responses for melanoma or renal cell carcinoma patients with only a few patients experiencing recurrence [4]. However, administered systemically at high doses, IL2 can cause severe toxicity including life-threatening side effects reducing the number of suitable patients and narrowing the therapeutic window. Different IL2 muteins [20] and fusion proteins [16] have addressed the limitations of high dose IL2 treatment in attempts to improve the therapeutic index. The *in silico* designed IL2 mimic, Neoleukin™-2/15, was developed by Baker and coworkers and is being explored by Neoleukin™ Therapeutics Inc. for its antitumor potential [37]. However, the therapeutic potential of the cytokine mimic might be limited by rapid renal clearance due to its small size and its lack of tumor-homing potential.

In this study, we have investigated if the therapeutic potential of the engineered cytokine can be improved by facilitating selective tumor accumulation. We have described the production of novel fusion proteins (L19-Neo™ and Neo™-L19) that bind EDB of fibronectin present on the tumor extracellular matrix. The activities of the diabody and cytokine moieties were characterized *in vitro*, and selective tumor accumulation was demonstrated in F9 tumor bearing mice. Since Neo™-L19 had superior tumor accumulation, it was investigated in two syngeneic mouse cancer models (F9 and WEHI-164) showing potent antitumor activity as monotherapy.

The targeting of cytokines to the tumor microenvironment by the means of antibody fusion can lead to selective tumor accumulation as observed for Neo™-L19 [13, 16, 46–48]. As expected, a fusion protein of irrelevant specificity in the mouse (Neo™-KSF) showed a reduced tumor uptake, confirming the role played by the L19 antibody in the active delivery of the payload (Supplementary Figure 4).

Bispecific antibodies carrying high-affinity binding moieties towards a tumor-associated antigen and the CD3 antigen on T cells exhibit an *in vivo* localization both to tumors and to secondary lymphoid organs [49]. It has previously been claimed that interactions between the cytokine moieties and their cognate receptors on circulating lymphocytes or in secondary lymphoid organs might hamper the tumor targeting properties of fusion proteins [40]. This feature has not been seen for many immunocytokines based on the L19 antibody [13, 50–52] and was also not observed for Neo™-L19. Indeed, the fusion protein exhibited a preferential uptake in the tumor (8.3 ± 2.2% ID/g) and a tumor: spleen ratio of approximately 16 twenty-four hours after intravenous administration. The absence of immunocytokine trapping on lymphocytes may be due to the low number of cognate receptors on T cells and NK cells, or to an insufficient kinetic stability of the cytokine: receptor complex. The diabody format, chosen in our work, may be preferable to the more conventional IgG format, since the reduced molecular weight and the absence of FcRn binding may lead to better penetration into the neoplastic mass and faster clearance from circulation [16]. Even though EDB (+)-fibronectin expression is typically associated with the tumor neo-vasculature, the antigen is predominantly found on the abluminal aspect of tumor blood vessels [53].

Mechanistic investigations into tumor targeted IL2 based treatments have indicated that immunological responses are correlated with an increased intratumoral presence of NK and CD8^+^ T cells [17, 54]. While importance of NK and CD8^+^ T cells have also been indicated for IL15 based treatments [55–57], these treatments have received considerable interest for their increased Effector: Treg ratio [58, 59], which is related to improved cancer therapies. Neoleukin™-2/15 cancer treatments have also been shown to increase the CD8^+^: Treg suggesting a mechanism similar to IL2 and IL15 [37]. We therefore believe that the mechanism for the antitumor activity of the immunocytokines is similar to these other cytokines, but must be further investigated in future studies.

Different immunocytokines containing IL2 or IL2 muteins are currently being investigated in the clinic to treat varies malignancies [41, 60–63]. For certain cytokines (such as IL2, IL12 and TNF) the fusion with a tumor-homing antibody allows the creation of novel biopharmaceuticals, which display a preferential uptake in tumor lesions and which perform substantially better than the corresponding nontargeted cytokine in immunocompetent mouse models [50, 51, 64, 65]. The binding of L19-Neo™ and Neo™-L19 to surface immobilized EDB indicated differences in the binding kinetics under circumstances that allowed for avid binding (Figure 1E, 1J). While the differences might impact the biodistribution of the immunocytokines, other factors will also have a major impact on the localization *in vivo*. We and others have reported that not all cytokine payloads can be efficiently delivered to the tumor by fusion with antibodies. Cytokine trapping at low dose has been reported for fusion proteins based on interferon-gamma [66, 67], but also for IL15 fusions [55, 68]. In other cases, aberrant glycosylation may lead to suboptimal biodistribution performance [2, 69, 70]. Neo™-L19 is not glycosylated and we were pleased to see how the L19-based targeted delivery of the engineered IL2 and IL15 mimic led to an improved therapeutic effect for the F9 teratocarcinoma model. The contribution of L19-based targeting to an increased therapeutic index was less striking in the WEHI-164 model. Collectively, the results presented indicate that a new fusion protein could be created, which displayed an excellent performance in biodistribution studies and which mediated a potent anticancer activity in two immunocompetent mouse models of cancer when used as single agent. The results suggest that this product, or similar fusion proteins featuring an N-terminal fusion in the diabody format, may deserve to be investigated in clinical trials.

## MATERIALS AND METHODS

### Design and cloning of the immunocytokines

The sequences for the constructs can be found in the Supplementary Information. The diabodies consist of a V_H_ and a V_L_ domain that are connected by a 5 amino acid (15 bp) linker. The diabody and the novel cytokine were connected by a 15 amino acid (45 bp) linker. In the 5′ of the protein gene, the sequence was linked to a mammalian signal peptide that allows for transient gene expression in CHO-S cells. The gene for L19-Neo™ was purchased from GenScript in a pcDNA3.1(+) vector, while the Neo™-L19 sequence cloned from the previous sequence and double digested with HindIII and EcoRI, and subsequently ligated into digested pcDNA3.1(+). The KSF construct was cloned by Gibson Isothermal assembly (NewEnglandBiolabs #E2621S) replacing the L19 moiety.

### Protein expression by transient gene expression

Transient gene expression was used to expressed all of the proteins. For L19-Neo™, Neo™-L19, His_6_-Neo™ and Neo™-KSF, CHO-S cells were diluted to a concentration of 4 × 10^6^ cells/mL in Pro-CHO medium (Lonza) containing 8 mM Ultraglutamine (Gibco), 4 mM HT supplement (Gibco), and 1% antibiotic-antimycotic (Gibco). The plasmid (0.8 μg/10^6^ cells) and PEI (2.5 μg/10^6^ cells) were added to the cells, and the mixture was incubated at 31 °C for 6 days for protein production. For hCD25, the plasmid DNA (1.25 μg/10^6^ cells) was diluted in sterile NaCl solution (150 mM, 25 μL/10^6^ cells). This solution was mixed with PEI (5 μg/10^6^ cells) in sterile NaCl solution (150 mM, 25 μL/10^6^ cells) and incubated at room temperature for 10 min. The PEI-DNA solution was mixed with CHO-S cells (2 × 10^6^ cells/mL) in Pro-CHO medium (Lonza) and incubated at 37°C for 4 hours. The culture was diluted 1:1 with PowerCHO medium (Lonza) and incubated at 31°C for 6 days.

The cells were removed by centrifugation, and the supernatant was filtered. The proteins were captured by protein A affinity (L19-Neo™, Neo™-L19, and Neo™-KSF) or Ni-NTA (His_6_-Neo™ and hCD25) chromatography. Proteins from protein A columns were eluted with TEA (1.4% V/V%) into tubes containing a neutralizing solution (Tris 1 M, pH 7.4), while the proteins from His-tag purification were eluted with imidazole (500 mM, PBS, pH 7.4). The products were dialyzed into PBS (pH 7.4) at 4°C. Biochemical analysis was performed by non-reducing and reducing SDS-PAGE with Coomassie Blue staining, size exclusion chromatography using a Superdex 200 Increased 10/300GL column (Flow rate: 0.75 mL/min, GE Healthcare) on an Äkta Pure FPLC system, and LC-MS (Column: 2.1 × 50 mm Acquity BEH300 C4 1.7 μm (Waters), Instrument: Waters Acquity UPLC H-Class 147 coupled with a Waters Xevo G2-X2 Qtof).

### Cell cultures

CHO-S cells were cultured in Power-CHO medium (Lonza), containing 8 mM Ultraglutamine (Lonza), 4 mM HT-supplement and 1× antibiotic-antimycotic (Gibco) at 37°C. The cells were maintained at densities between 0.5 and 8.0 × 10^6^ cells/mL in a shaking incubator (150 rpm).

In general, the remaining cancer cells were cultured under a 5% CO_2_ atmosphere at 37°C. CTLL-2 and transduced reporter CTLL-2 cells [45] were cultured in RPMI (Gibco) containing 10% FBS (Gibco), 1× antibiotic-antimycotic (Gibco), 2 mM Ultraglutamine (Lonza), 25 mM HEPES (Gibco), 50 μM β-mercaptoethanol (Sigma) and 60 U/mL IL-2 (Proleukin, Roche Diagnostics). F9 teratocarcinoma cells were cultured in flasks coated with 0.1% gelatin (Type B from Bovine Skin, Sigma), and they were cultured in DMEM (Gibco) containing 10% FBS (Gibco) and 1× antibiotic-antimycotic (Gibco). WEHI-164 fibrosarcoma cells were cultured in RPMI (Gibco) containing 10% FBS (Gibco) and 1× antibiotic-antimycotic (Gibco).

### Surface plasmon resonance

All surface plasmon resonance experiments were performed on a Biacore S200 instrument at 25°C. The binding events were investigated in PBS buffer (Gibco) containing 0.005% Tween-20. The proteins were immobilized on CM-5 S-series (GE Healthcare) sensor chips by activation of the surface with EDC and NHS. The remaining activated esters were capped with ethanolamine.

For EDB binding, EDB was captured on the NHS activated surface to a level of approximately 620 RU. The reference flow was capped with ethanolamine. A 2-fold dilution series of the immunocytokines (500–31.25 nM) were injected for 180 sec (association) and followed by 600 sec of dissociation at a flow rate of 20 μL/min. The surface was regenerated with five 15 sec injections of 10 mM HCl.

For the CD25 binding, CD25 was captured on the NHS activated surface to a level of approximately 550 RU. The immunocytokines were injected in a 2-fold dilution series (500 nM–31.25 nM) with 90 sec association and 300 sec dissociation at a flow rate of 30 μL/min. The surface was regenerated with a 15 sec injection of 1 M MgCl_2_ in 10 mM sodium acetate pH 5.5.

### Binding analysis by flow cytometry

CTLL-2 cells were washed twice with HBSS (Gibco) and incubated in culturing medium without IL2 for 24 h. The starved CTLL-2 cells were centrifugated and resuspended in FACS buffer (0.5% BSA, 2 mM EDTA in PBS) to a density of 3 × 10^6^ cells/mL. The cells were seeded in a 96 well plate containing 300,000 cells/well.

For staining procedures involving FITC-modified immunocytokines, proteins were incubated with cells on ice for 1 h. The cells were then washed twice with FACS buffer, resuspended in FACS buffer, and analyzed by flow cytometry (CytoFLEX, Beckman Coulter). The data was analyzed by the software FlowJo (v3, Tree Star).

### Proliferation assay

CTLL-2 cells were washed twice with HBSS (Gibco) and incubated in culturing medium without IL2 for 24 h. The IL2-starved cells were seeded in a 96-well plate with a density of 25,000 cells/well. The cells were incubated in medium, containing the immunocytokines at varying concentration, at 37°C under 5% CO_2_ for 48 h. 20 μL CellTiter 96 Aqueous One Solution (Promega) was added to each well and the plate incubated for 1–2 hours at 37°C. The absorbance was measured at 490 nm and 620 nm. The relative proliferation was calculated as: relative proliferation = (OD490-620treated-OD490-620medium)/(OD490-620untreated-OD490-620medium). The data was fitted in GraphPad Prism 7 using a [Agonist] vs response (three parameters) model.

### NF-κB activity assay

The NF-κB reporter cell line was used according to the previous report [45]. Briefly, the reporter cells (transduced reporter CTLL-2) were starved for IL2 6-9 hours prior to use. The cells were seeded in 96-well plates to a density of 50,000 cells/well. The cells were incubated in growth medium (200 μL), containing the immunocytokines in varying concentrations, at 37°C under a 5% CO_2_ atmosphere for several hours. The luciferase production was assessed by transferring 20 μL of the solution to an opaque 96-well plate (Optiplate-96, white, Perkin Elmer). To each well was added 80 μL 1 μg/mL Coelenterazine (Carl Roth AG) in PBS, and the luminescence (595 nm) was measured immediately after the addition of the reagent. The relative luminescence was calculated by division with values obtained for cells cultured without inducer added to the medium. The data was fitted in GraphPad Prism 7 using a [Agonist] vs response (three parameters) model.

### FITC labelling of fusion proteins

The proteins were buffer exchanged into a carbonate buffer (100 mM, pH 9.0) by washing in Amicon Ultra ultracentrifugation filters (MWCO 10k, 11000 g, 10 min - Sigma) three times. To the solution of protein was added 50 molar equivalents of FITC (Sigma), and the reaction mixture was incubated at 4°C overnight. The conjugate was purified using a PD SpinTrap™ G-25 (Sigma) according to Manufacturer’s instructions.

### Animal experiments

7–8 weeks old immunocompetent female BALB/c mice and 7–8 weeks old immunocompetent female 129/Sv were purchased from Janvier (France). On the day of tumor injections, the exponentially growing tumor cells (F9 teratocarcinoma or WEHI-164 fibrosarcoma) were harvested, washed and resuspended in Hank’s Balanced Salt Solution, HBSS, (Gibco). The tumor cells (F9 cells; 10 × 10^6^ cells per mouse for 129/Sv mice. WEHI-164 cells; 2.5 × 10^6^ cells per mouse for BALB/c mice) were injected subcutaneously in the right flank. The animals were monitored daily and the tumor sizes were measured with a caliper. Tumor volume was calculated as: Tumor volume [mm^3^] = length [mm] × width [mm] × width [mm] × 0.5. The animals were euthanized when the longest diameter of the tumor exceeded 15 mm, weight loss was greater than 15% body weight, tumors ulcerated, or 24 h after the last injection in biodistribution experiments. For the biodistribution experiments, tumor, lung, liver, spleen, heart, kidney, intestine, tail, and blood were collected.

All animal experiments were performed under the project license ZH04/2018 granted by the Veterinäramt des Kanton Zürich, Switzerland, in agreement with Swiss regulations. The animals were housed (groups of 2–5 mice per cage with food and water ad libitum) in an open hygiene barrier (reduced pathogen exposure) facility at the Swiss Federal Institute of Technology (ETH Zurich).

### Quantitative biodistribution studies

Chloramine T hydrate (5 μL, 5 mg/mL in MQ water) was added to a solution of immunocytokines (100 μg) and iodine-125 (200 μCi, Hartmann Analytic). After 1 min and 45 sec, the radiolabeled proteins were purified by size exclusion chromatography using PD-10 columns (GE Healthcare) blocked with BSA (1 mL of 1 mg/mL in sterile filtered PBS) and equilibrated with sterile filtered PBS (25 mL). Immunocompetent 129/Sv mice (*n* = 3) were injected subcutaneously in the right flank with 10 × 10^6^ F9 teratocarcinoma cells. The mice were monitored daily, and the tumor sizes were measured with a caliper. Tumor volume was determined as previously described. When the tumors had a volume of approximately 200–300 mm^3^, 10 μg of radioiodinated immunocytokine was injected intravenously into the lateral tail vein. The mice were sacrificed after 24 h, and the tumor, lung, liver, spleen, heart, kidney, intestine, tail, and blood were collected. The radioactivity was quantified by a Packard Cobra γ-counter. The radioactivity was expressed as a percentage of the injected dose per gram of tissue (%ID/g) ± SD.

### Therapy experiments

On the day of tumor injections, the exponentially growing tumor cells (F9 teratocarcinoma or WEHI-164 fibrosarcoma) were harvested by trypsin digestion, washed and resuspended in HBSS (Gibco). The tumor cells (F9 cells; 10 × 10^6^ cells per mouse for 129/Sv mice. WEHI-164 cells; 2.5 × 10^6^ cells per mouse for BALB/c) were injected subcutaneously in the right flank. The animals were monitored daily and the tumor sizes were measured with a caliper. Tumor volumes were calculated as previously described. When the tumors were clearly detectable with an average size of about 90 mm^3^, the mice were randomly divided into the different treatment groups (*n* = 4–5). The treatments were initiated at an average tumor size of approximately 90 mm^3^ and were administered intravenously into the lateral tail vein. For the F9 teratocarcinoma therapies, the mice received three injections of 10, 60, or 100 μg of Neo™-L19, 60 or 100 μg Neo™-KSF or phosphate buffered saline at intervals of 48 h. For the WEHI-164 fibrosarcoma, the mice received two injects of 100 μg Neo™-L19, 100 μg Neo™-KSF or 37 μg (equimolar with the immunocytokines) His_6_-Neo™ or phosphate buffered saline with a 48 h interval. Animals were euthanized according to the conditions previously described. Animals with complete responses were monitored for an additional 60 days after last tumor measurement to investigate tumor regrowth.

## SUPPLEMENTARY MATERIALS




