# Supplementary material for: Targeting an engineered cytokine with interleukin-2 and interleukin-15 activity to the neovasculature of solid tumors
Source: Oncotarget. 2020 Nov 3;11(44):3972–83. doi: 10.18632/oncotarget.27772 (PMC7646832; doi:10.18632/oncotarget.27772)
Supplement: Supplementary file 1 [file oncotarget-11-3972-s001.pdf]

Targeting an engineered cytokine with interleukin-2 and interleukin-15 activity to the neovasculature of solid tumors

SUPPLEMENTARY MATERIALS

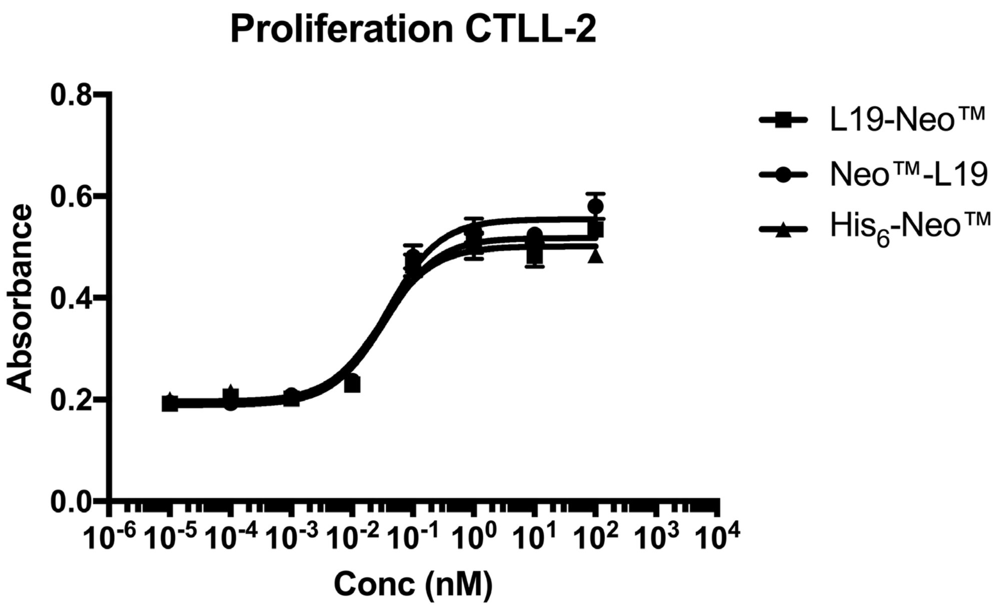

Supplementary Figure 1: Proliferation assay of immunocytokines using murine CTLL-2 cells. EC50 (mean (n = 3) ± SD): L19-Neo™ 0.035 ± 0.007 nM, Neo™-L19 0.038 ± 0.007 nM, His^6-Neo™ 0.029 ± 0.008 nM.

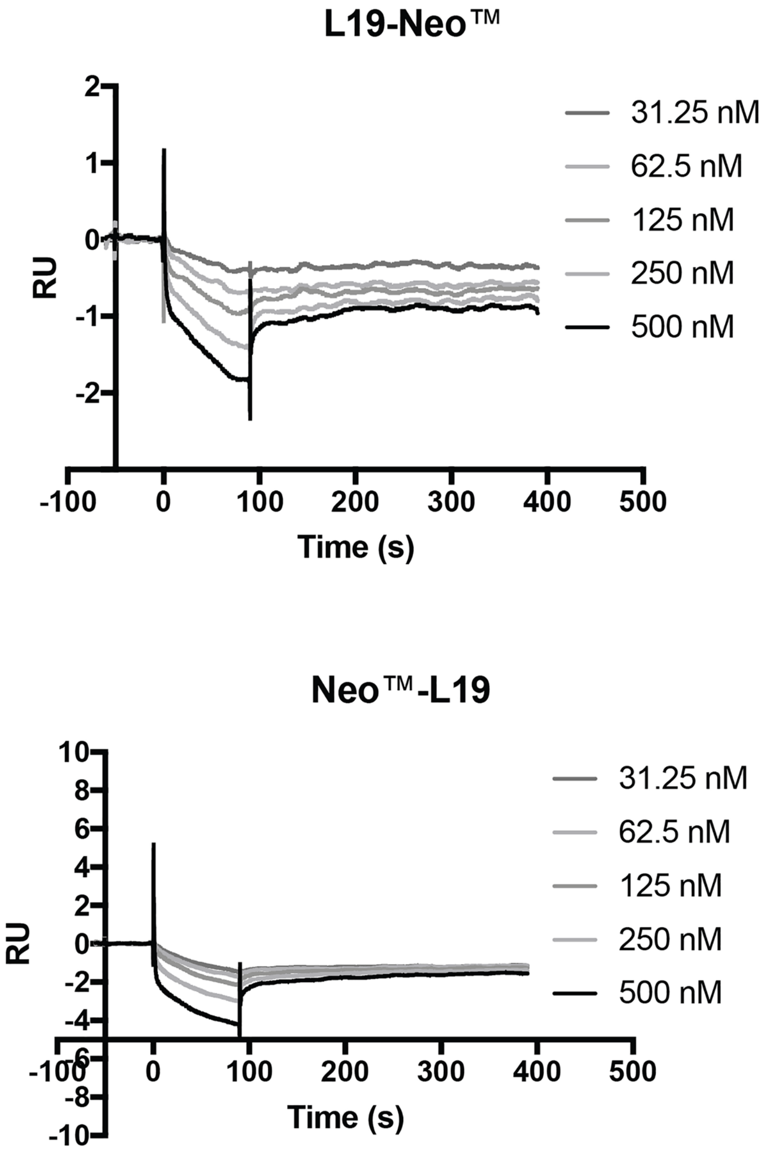

Supplementary Figure 2: Binding characterization between the fusion proteins and surface immobilized hCD25. The figure depicts the experiments from Figure 2C zoomed on the y-scales.

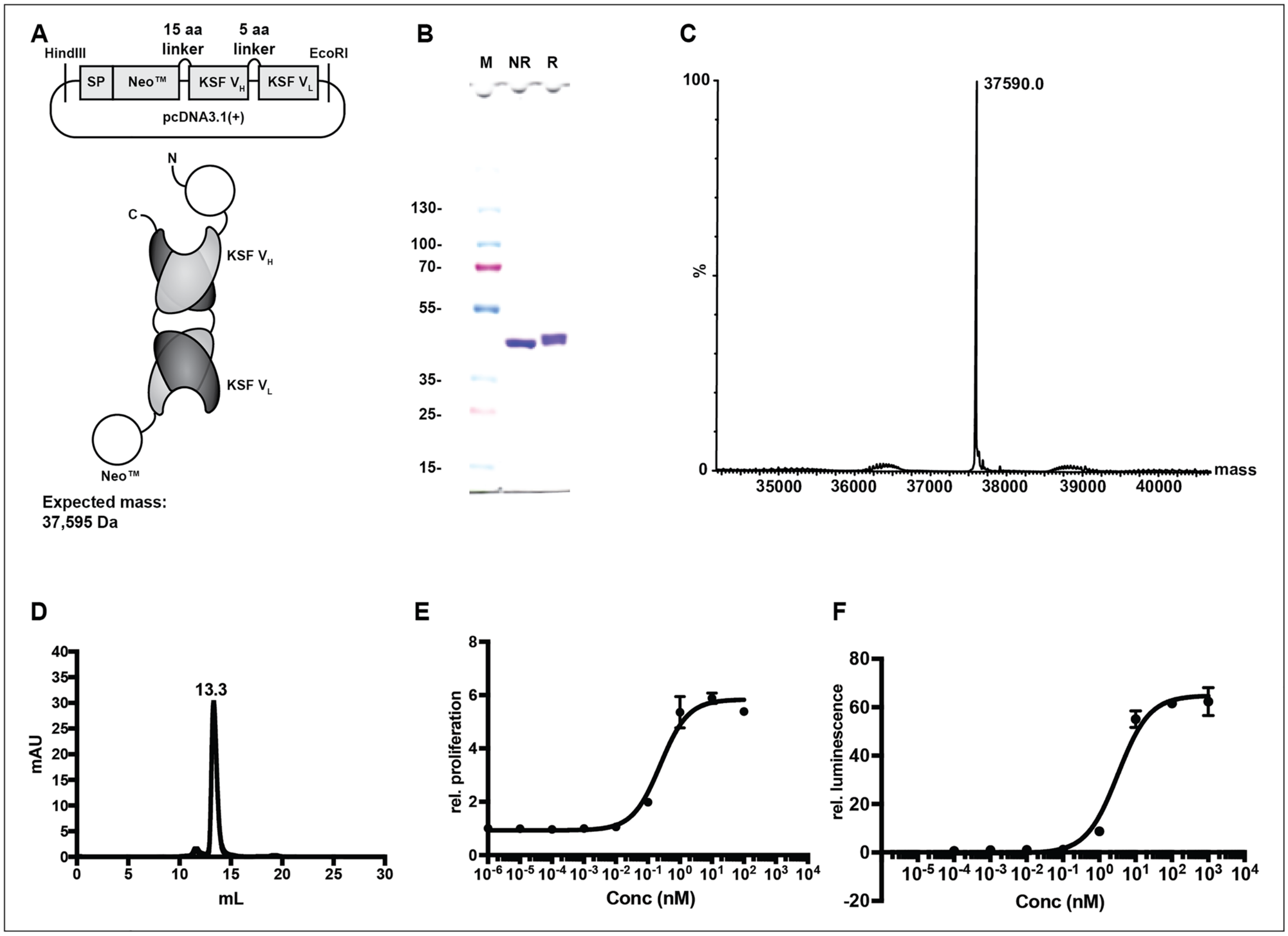

Supplementary Figure 3: Design and *in vitro* characterization of Neo™-KSF. (**A**) Cloning scheme, expected protein structure and expected mass. (**B**) SDS-PAGE analysis stained for protein with Coomassie Blue. M: PageRuler™ Plus Prestained Protein Ladder, NR: non-reducing, R: reducing. (**C**) MS profile. (**D**) Size exclusion chromatography analysis. (**E**) Proliferation assay with murine CTLL-2 cells using different concentrations of Neo™-KSF. The experiments were performed in triplicates and the error bars are equal to the standard deviation. EC50 (mean (n = 3) ± SD): Neo™-KSF 0.244 ± 0.044 nM. (**F**) NF-κB reporter assay for Neo™-KSF. The experiments were performed in triplicates and the error bars are equal to the standard deviation. EC50 (mean (n = 3) ± SD): Neo™-KSF 3.25 ± 0.58 nM.

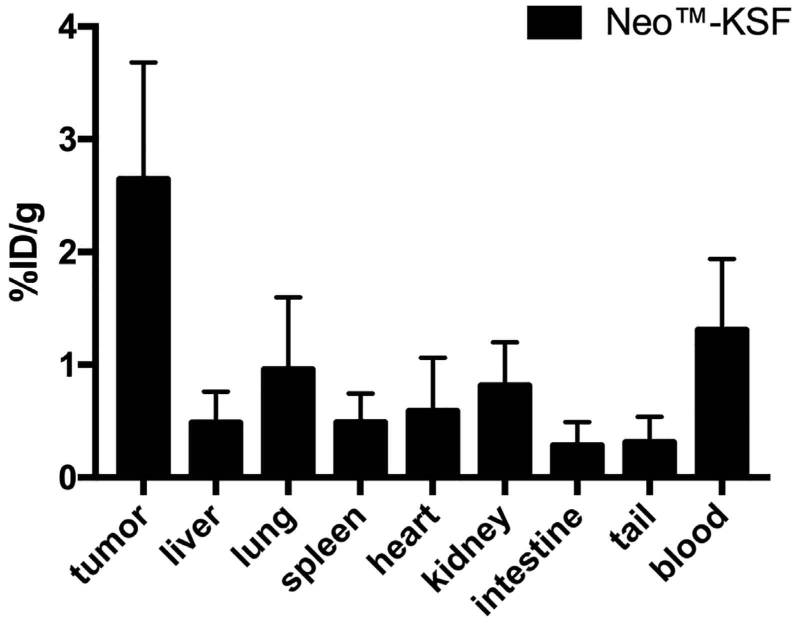

Supplementary Figure 4: Quantitative biodistribution experiment of Neo™-KSF using intravenously injected radioiodinated fusion protein. The analysis was performed after 24 h and is expressed in percentage of the injected dose per gram of tissue (%ID/g ± SD, *n* = 3).

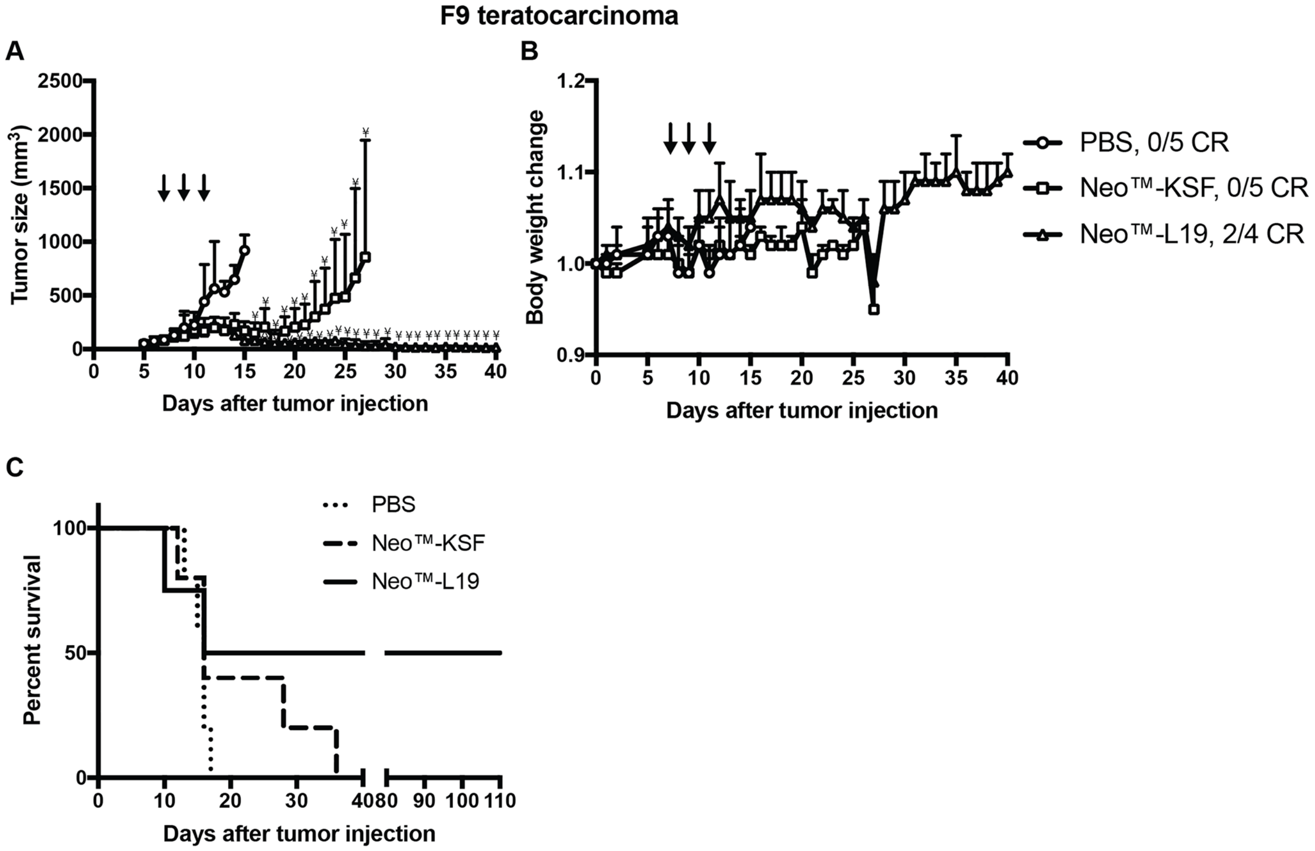

Supplementary Figure 5: Activity of Neo™-L19 and Neo™-KSF in F9 teratocarcinoma tumor bearing mice. (**A**) Tumor bearing mice received 3 injections (↓, days 7, 9 and 11) of Neo™-L19 (60 μg per inj), Neo™-KSF (60 μg per inj) or saline (PBS) when the tumors reached an average size of approximately 85 mm^3. The data is represented as the mean ± SD. Statistical analysis was performed by two-way ANOVA with post Bonferroni test (Not significant (ns) $P > 0.05$, $^{*}P < 0.05$, $^{**}P <. 0.01$, $^{***}P < 0.001$, $^{****}P < 0.0001$). On day 15, the immunocytokine treatments were different from the saline treatment ($^{****}P < 0.0001$). On day 27, the Neo™-L19 treatment was different from the Neo™-KSF treatment ($^{***}P < 0.001$, only 2 mice in each group). (**B**) Body weight changes during the treatment represented as the mean ± SD. (**C**) Survival plot.

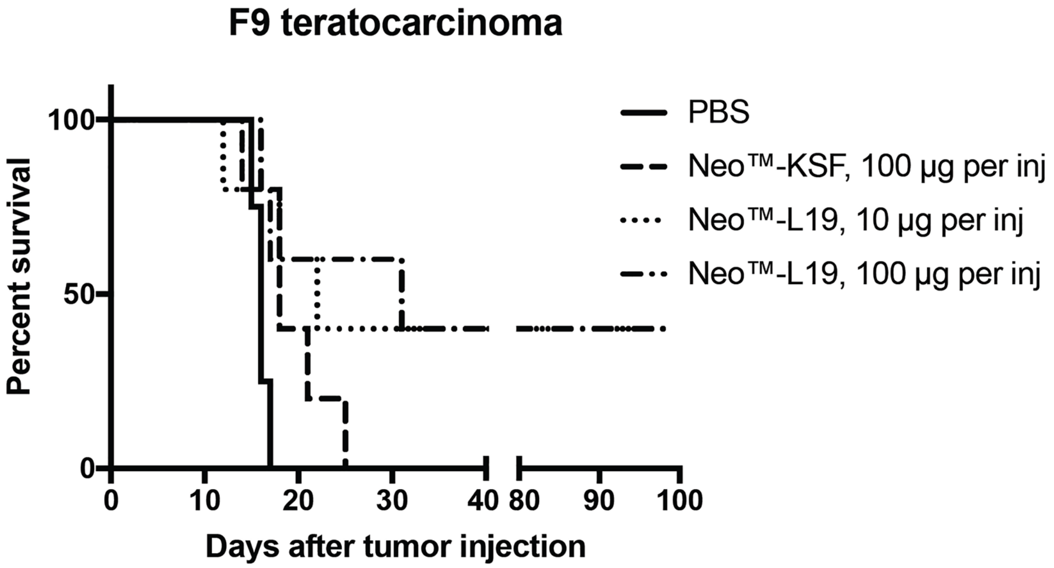

Supplementary Figure 6: Additional information for the F9 teratocarcinoma therapy shown in Figure 4. Survival plot.

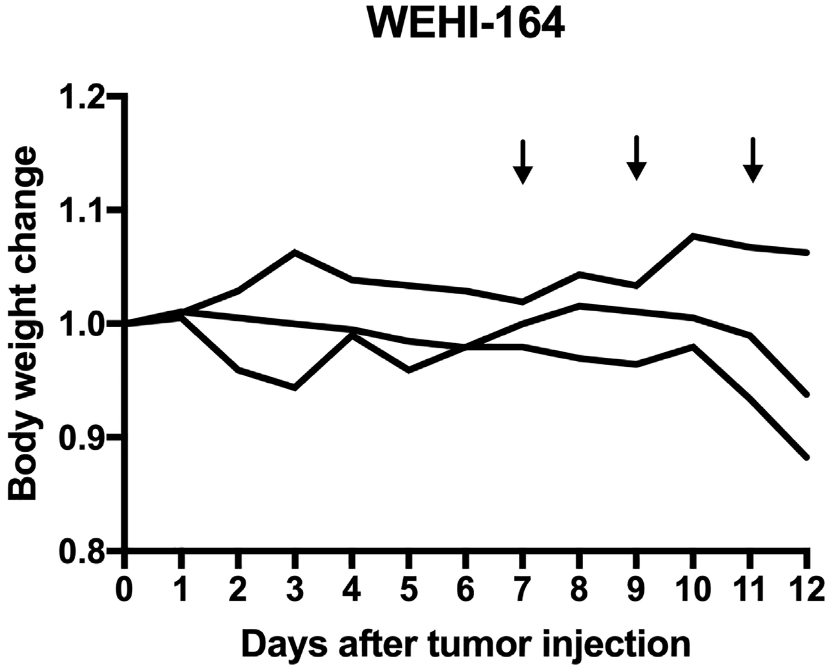

Supplementary Figure 7: Initial investigation of the treatment tolerability in mice bearing WEHI-164 fibrosarcomas. Tumor bearing mice received 3 injections (↓) of Neo™-L19 (100 µg per inj) when the tumors reached a size of 90 mm^3. Each curve represents a single animal.

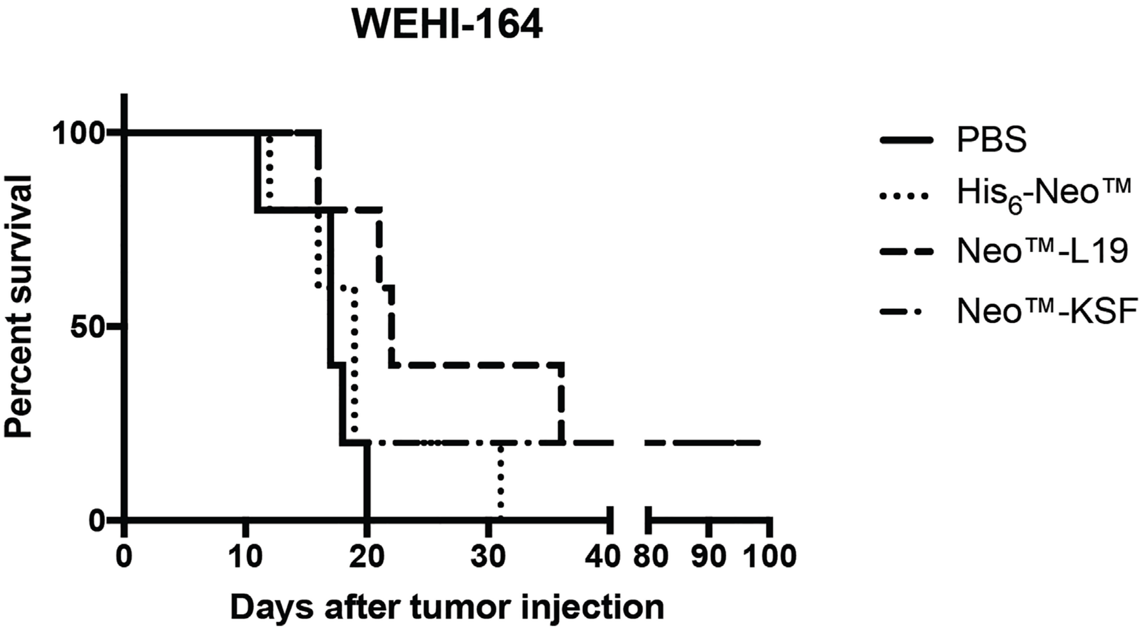

Supplementary Figure 8: Additional information for the WEHI-164 fibrosarcoma therapy shown in Figure 4. Survival plot.
