# Supplementary material for: Targeting an engineered cytokine with interleukin-2 and interleukin-15 activity to the neovasculature of solid tumors
Source: Oncotarget. 2020 Nov 3;11(44):3972–83. doi: 10.18632/oncotarget.27772 (PMC7646832; doi:10.18632/oncotarget.27772)
Supplement: Supplementary file 2 [file oncotarget-11-3972-s002.pdf]

Sequence Information

L19-Neo™

Protein: Secretion - L19 – Linker - CleavLeft - Neo™

PSLSTMGWSLILLFLVAVATGVHSEVQLLESGGGLVQPGGSLRLSCAASGFTFSSFSMSWVRQAPGKGLEWVSSISGSSGTTYY
ADSVKGRFTISRDNSKNTLYLQMNSLRAEDTAVYYCAKPFPYFDYWGQGTLVTVSSGSSGGEIVLTQSPGTLSLSPGERATLSCRASQ
SVSSSFLAWYQQKPGQAPRLLIYYASSRATGIPDRFSGSGSGTDFTLTISRLEPEDFAVYYCQQTGRIPPTFGQGTKVEIKSSSSGSSSS
GSSSSGSHMPKKKIQLHAEHALYDALMILNIVKTNSPPAEEKLEDYAFNFELILEEIARLFESGDQKDEAEKAKRMKEWMKRIKTTASE
DEQEEMANAIITILQSWIFS

DNA: HindIII-Signal peptide- L19- Linker-CleavLeft- Neo™- STOP - EcoRI

CTCCAAGCTTGTCGACCATGGGCTGGAGCCTGATCCTCCTGTTCCTCGTCGCTGTGGCTACAGGTGTGCACTCG*gaggtgcagctg*
ttggagtctgggggaggcttggtacagcctggggggtccctgagactctcctgtgcagcctctggattcacctttagcagtttttcgatgagctgggtccgccagg
ctccagggaaggggctggagtgggtctcatctattagtggtagttcgggtaccacatactacgcagactccgtgaagggccggttcaccatctccagagacaat
*tccaagaacacgctgtatctgcaaatgaacagcctgagagccgaggacacggccgt*atattactgtgcgaaaccgtttccgtattttgactactggggccaggga
accctggtcaccgtctcgagt*gggtccagtggcggt*gaaattgtgttgacgcagtctccaggcaccctgtctttgtctccaggggaaagagccaccctctcctgcagg
gccagtcagagtgttagcagcagctttttagcctggtaccagcagaaacctggccaggctcccaggctcctcatctattatgcatccagcagggccactggcatcccag
acaggttcagtggcagtgggtctgggacagacttcactctcaccatcagcagactggagcctgaagattttgcagtgtattactgtcagcagacgggtcgtattccgcc
gacgttcggccaagggaccaaggtggaaatcaaaTCTTCCTCATCGGGTAGTAGCTCTTCCGGCTCATCGTCCAGCGGCagccatatgCCTAA
GAAGAAAATTCAGCTGCACGCAGAACACGCTCTGTATGACGCTCTGATGATCCTGAACATCGTCAAAACTAACTCACCTCCTGCCG
AGGAGAAGCTGGAGGACTACGCTTTCAACTTTGAGCTGATCCTGGAGGAGATCGCCAGGCTGTTCGAGTCCGGCGACCAGAAGGAT
GAGGCCGAGAAGGCTAAGAGGATGAAGGAGTGGATGAAGCGGATCAAGACCACAGCTAGCGAGGACGAACAGGAGGAGATGGCAAA
CGCTATTATCACTATTCTGCAGTCTTGGATTTTCAGCTAGCTTAATGAGAATTCGCGGGG

Neo™-L19

Protein: Secretion-Cleavleft - Neo™ – linker – L19

PSLSTMGWSLILLFLVAVATGVHSGSHMPKKKIQLHAEHALYDALMILNIVKTNSPPAEEKLEDYAFNFELILEEIARLFESGDQK
DEAEKAKRMKEWMKRIKTTASEDEQEEMANAIITILQSWIFSSSSSGSSSSGSSSSGEVQLLESGGGLVQPGGSLRLSCAASGFTFSSF
SMSWVRQAPGKGLEWVSSISGSSGTTYYADSVKGRFTISRDNSKNTLYLQMNSLRAEDTAVYYCAKPFPYFDYWGQGTLVTVSSGS
SGGEIVLTQSPGTLSLSPGERATLSCRASQSVSSSFLAWYQQKPGQAPRLLIYYASSRATGIPDRFSGSGSGTDFTLTISRLEPEDFAVYY
CQQTGRIPPTFGQGTKVEIK

DNA: HindIII-Signal peptide-CleavLeft -Neo™ - Linker - L19- STOP - EcoRI

CTCCAAGCTTGTCGACCATGGGCTGGAGCCTGATCCTCCTGTTCCTCGTCGCTGTGGCTACAGGTGTGCACTCGggcagccat
atgCCTAAGAAGAAAATTCAGCTGCACGCAGAACACGCTCTGTATGACGCTCTGATGATCCTGAACATCGTCAAAACT
AACTCACCTCCTGCCGAGGAGAAGCTGGAGGACTACGCTTTCAACTTTGAGCTGATCCTGGAGGAGATCGCCAGGCTGTTCGAGTC
CGGCGACCAGAAGGATGAGGCCGAGAAGGCTAAGAGGATGAAGGAGTGGATGAAGCGGATCAAGACCACAGCTAGCGAGGACGAAC
AGGAGGAGATGGCAAACGCTATTATCACTATTCTGCAGTCTTGGATTTTCAGCTCTTCCTCATCGGGTAGTAGCTCTTCCGGATCC
TCGTCCAGCGGC*gaggtgcagctgttggagtctgggggaggcttggtacagcctggggggtccctgagactctcctgtgcagcctctggattcacctttagc*
agtttttcgatgagctgggtccgccaggctccagggaaggggctggagtgggtctcatctattagtggtagttcgggtaccacatactacgcagactccgtgaa
*gggccggttcaccatctccagagacaattccaagaacacgctgtatctgcaaatgaacagcctgagagccgaggacacggccgt*atattactgtgcgaaaccg
tttccgtattttgactactggggccagggaaccctggtcaccgtctcgagt*gggtccagtggcggt*gaaattgtgttgacgcagtctccaggcaccctgtctttgtctcc
aggggaaagagccaccctctcctgcagggccagtcagagtgttagcagcagctttttagcctggtaccagcagaaacctggccaggctcccaggctcctcatctatta
tgcatccagcagggccactggcatcccagacaggttcagtggcagtgggtctgggacagacttcactctcaccatcagcagactggagcctgaagattttgcagtgta
ttactgtcagcagacgggtcgtattccgccgacgttcggccaagggaccaaggtggaaatcaaaTAGCTTAATGAGAATTCGCGGGG

Neo™-KSF

protein seq: Cleavleft - Neo™ – linker – KSF

GSHMPKKKIQLHAEHALYDALMILNIVKTNSPPAEEKLEDYAFNFELILEEIARLFESGDQ
KDEAEKAKRMKEWMKRIKTTASEDEQEEMANAIITILQSWIFSSSSSGSSSSGSSSSGEVQ
LLESGGGLVQPGGSLRLSCAASGFTFSSYAMSWVRQAPGKGLEWVSAISGSGGSTYYA
DSVKGRFTISRDNSKNTLYLQMNSLRAEDTAVYYCAKSPKVSLFDYWGQGTLVTVSSG
GSGGSELTQDPAVSVALGQTVRITCQGDSLRSYYASWYQQKPGQAPVLVIYGKNNRPS
GIPDRFSGSSSGNTASLTITGAQAEDEADYYCNSSPLNRLAVVFGGGTKLTVLG

DNA: HindIII-Signal peptide-CleavLeft -Neo™ - Linker - KSF- STOP - EcoRI

CTCCAAGCTTGTCGACCATGGGCTGGAGCCTGATCCTCCTGTTCCTCGTCGCTGTGGCTACAGGTGTGCACTCGggcagccat
atgCCTAAGAAGAAAATTCAGCTGCACGCAGAACACGCTCTGTATGACGCTCTGATGATCCTGAACATCGTCAAAACT
AACTCACCTCCTGCCGAGGAGAAGCTGGAGGACTACGCTTTCAACTTTGAGCTGATCCTGGAGGAGATCGCCAGGCTGTTCGAGTC
CGGCGACCAGAAGGATGAGGCCGAGAAGGCTAAGAGGATGAAGGAGTGGATGAAGCGGATCAAGACCACAGCTAGCGAGGACGAAC
AGGAGGAGATGGCAAACGCTATTATCACTATTCTGCAGTCTTGGATTTTCAGCTCTTCCTCATCGGGTAGTAGCTCTTCCGGATCC
TCGTCCAGCGGCGAGGTGCAGCTGTTGGAGTCTGGGGGAGGCTTGGTACAGCCTGGGGGGTCCCTGAGAC
TCTCCTGTGCAGCCTCTGGATTCACCTTTAGCAGCTATGCCATGAGCTGGGTCCGCCAGGCTCCAGGGA
AGGGGCTGGAGTGGGTCTCAGCTATTAGTGGTAGTGGTGGTAGCACATACTACGCAGACTCCGTGAAG
GGCCGGTTCACCATCTCCAGAGACAATTCCAAGAACACGCTGTATCTGCAAATGAACAGCCTGAGAGC
CGAAGACACGGCCGTATATTACTGTGCGAAATCGCCTAAGGTGTCGCTTTTTGACTACTGGGGCCAGG
GAACCCTGGTCACCGTCTCGAGTggcggtagcggagggTCTGAGCTGACTCAGGACCCCGCTGTGTCTGTGGC
CTTGGGACAGACAGTCAGGATCACATGCCAAGGAGACAGTCTCAGAAGCTATTATGCAAGCTGGTACC
AGCAGAAGCCAGGACAGGCCCCTGTACTTGTCATCTATGGTAAAAACAACCGGCCCTCAGGGATCCCA
GACCGATTCTCTGGCTCCAGCTCAGGAAACACAGCTTCCTTGACCATCACTGGGGCTCAGGCGGAAGA
TGAGGCTGACTATTACTGTAACTCCTCTCCCCTGAATCGGCTGGCTGTGGTATTCGGCGGAGGGACCA
AGCTGACCGTCCTAGGCTAGCTTAATGAGAATTCGCGGGG
